# Impact of moderate alcohol consumption on visuo-motor skills in neurosurgical residents

**DOI:** 10.1038/s41598-026-58320-5

**Published:** 2026-06-16

**Authors:** Anna Holzer, Marvin Darkwah Oppong, Lisa Schock, Ulrich Sure, Hendrik Schoell, Benno Hartung, Laurèl Rauschenbach

**Affiliations:** 1 Institute of Legal Medicine, University Hospital Essen, University Duisburg-Essen, Essen, Germany; 2 Department of Neurosurgery and Spine Surgery, University Hospital Essen, University Duisburg-Essen, Essen, Germany; 3 Center for Translational Neuro- and Behavioral Sciences (C-TNBS), University Duisburg-Essen, Essen, Germany; 4 Department of Plastic and Aesthetic Surgery, Red Cross Hospital Munich, Munich, Germany

**Keywords:** Alcohol, Neurosurgery, Coordination, Impairment, Blood alcohol concentration, Medical research, Neurology, Neuroscience

## Abstract

Alcohol adversely affects cognitive and motor functions that are critical for surgical performance. Although its general physiological and neurocognitive effects are well documented, evidence regarding its impact on complex, high-precision tasks—such as surgical procedures—remains limited. In this study, nine neurosurgeons performed a series of standardized surgical tasks assessing fine motor skills, gross motor control, and hand–eye coordination under three conditions: sober, acutely influenced (blood alcohol concentration [BAC] ~ 1.0 g/kg), and during the elimination phase several hours after alcohol consumption (BAC ~ 0.6 g/kg), with blood samples collected in each condition for BAC determination in accordance with German forensic standards. Across all nine participants, a total of 189 tests were conducted and evaluated, taking into account both the time required to complete each task and the quality of task performance. Task quality declined in both the acutely influenced and elimination phases. Significant impairments in quality were observed in almost all tasks. During acute influence, tasks such as suturing took longer to complete, whereas others like drilling were performed more quickly. In the elimination phase, all tasks were completed faster than at baseline. These findings suggest that even moderate alcohol intake can have lasting effects on surgical precision and may have implications for patient safety in operative settings.

**Supplementary Information:**

The online version contains supplementary material available at 10.1038/s41598-026-58320-5.

## Introduction

Alcohol consumption remains a major public health concern worldwide. According to the World Health Organization, an estimated 400 million individuals aged 15 years and older live with alcohol use disorders, and an estimated 209 million meet the criteria of alcohol dependence—with the highest prevalence in the European Region^1^. Germany’s per capita consumption of pure alcohol stands at 10.6 L annually, placing it among the highest in Europe^2^.

Physicians—particularly those working in high-stress specialties such as surgery—may be at increased risk for substance misuse. A German survey reported that 23% of physicians consumed alcohol at levels considered hazardous to health^3^. In other countries, rates of problematic alcohol use among physicians have been reported as high as 35%^4,5^, including a prevalence of alcohol abuse or dependence of 15.4% among American surgeons^6^. Several factors, such as young age, childlessness, and female sex, have been identified as potential contributors to this elevated risk^3,5,6^.

Occasional reports of surgeons performing procedures under the influence of alcohol, sometimes with substantial impairment, periodically draw public and media attention (e.g.,^7^). Although the topic of acute or residual alcohol effects on surgical performance remains largely taboo within the medical community, it warrants closer scientific examination. Unlike driving, no evidence-based thresholds exist to define fitness for surgery, and a zero-alcohol expectation is generally assumed. Nevertheless, surgeons who consumed alcohol the evening before often have little awareness of their potential residual blood alcohol concentration the following day.

Experimental studies have attempted to quantify the effects of alcohol on surgical performance. Van Dyken et al.^8^ found that even moderate alcohol consumption significantly impaired laparoscopic performance, with effects extending into the next day. Previous studies demonstrated measurable impairments in surgical^9^ and neurosurgical^10,11^ task performance under the influence of alcohol, especially with regard to fine motor skills and coordination. To date, only a single study has specifically examined the effects of alcohol on defined neurosurgical tasks^10^.

The present study therefore aims to explore task performance across a range of surgical and neurosurgical procedures under different blood alcohol concentrations (BAC) in a cohort of neurosurgeons of different training stages, to better understand the interaction between alcohol, motor skill integrity, and professional training.

## Material and methods

### Study cohort

The study was conducted among neurosurgeons at the University Hospital Essen between September 2024 and January 2025. Alcohol abstinence was confirmed prior to the trial via breath alcohol testing with a Draeger Alcotest device, and drug abstinence was verified through urine drug screening. To assess alcohol consumption behavior, the validated Alcohol Use Disorders Identification Test (AUDIT) was administered. Participants reporting alcohol intake exceeding two drinks per occasion on multiple days per week were categorized as at-risk drinkers.

### Study design

Participants underwent a controlled alcohol consumption trial. The target BAC of 1.0 g/kg was calculated individually based on self-reported height and weight using the Widmark formula^12^. Alcohol (beer or wine) was consumed within 1.5 h followed by an additional 0.5 h of resorption time. Light food (e.g. sandwiches, fruit) was available ad libitum throughout the study period to reduce potential confounding effects of fasting (especially alcohol-induced hypoglycaemia). The food intake was not systematically monitored.

Each participant completed seven task stations in randomized order. First, all participants were allowed to familiarize themselves with the tests and complete all tasks without any recording of time or performance quality. Subsequently, all tasks were performed under three conditions: sober (baseline, T0), during acute influence of alcohol (~ 1.0 g/kg BAC, T1), and during elimination phase (0.5–0.7 g/kg BAC, T2), approximately two to three hours after peak consumption and four to five hours after onset of alcohol intake. Immediately after participants had completed all tasks within each experimental condition, blood samples were collected by venipuncture for BAC determination.

### Task description

**Fig. 1:**
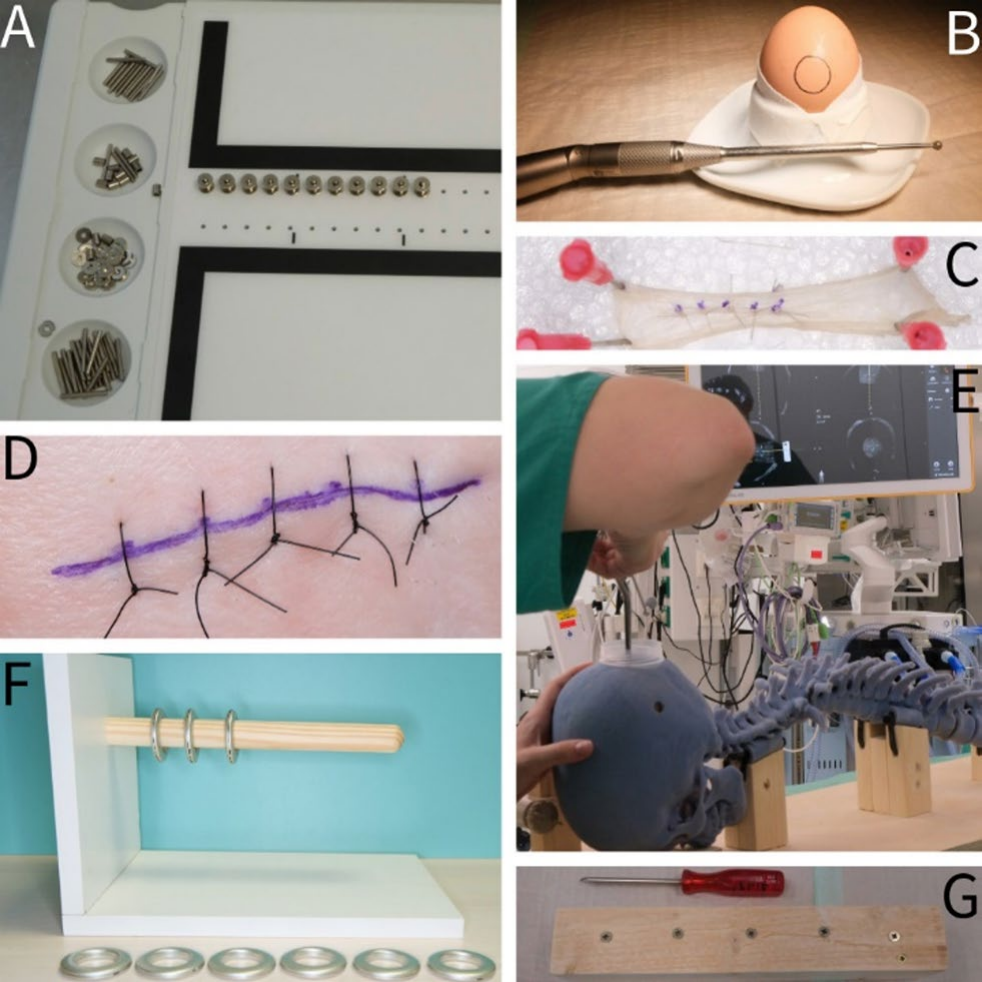
Representative images of the task stations included in the study design. (**A**) Perdue Pegboard. (**B**) Drilling. (**C**) Microsurgical suturing. (**D**) Macroscopic suturing. (**E**) Neuronavigation. (**F**) Curtain rod. (**G**) Screw removal.

Representative images illustrating each test are shown in Fig. 1.

#### General surgical tasks

- Macroscopic Suturing: A 6 cm incision was made through skin and subcutaneous tissue on a pigskin sample. Five suture points were marked at 1 cm intervals. Using vicryl and polyethylene sutures, participants independently performed both subcutaneous and cutaneous single-knot sutures. Time to completion, suture sufficiency and suture quality on a 5-point scale were assessed.
- Microsurgical Suturing: A segment of pig intestine was fixed on styrofoam. A 3 cm incision was made and five 0.5 cm–spaced suturing points marked. Under microscopic magnification, participants performed single-knot microsurgical sutures using vicryl. Time, suture sufficiency and suture quality (on a 5-point scale) were recorded.
- Screw Removal: Five 2 cm wood screws were inserted into a wooden block. Participants removed them using a standard manual screwdriver. Time, precision and procedural quality (number of incorrect placements of screwdriver tip, slipping or tool dropping) were evaluated.

#### Neurosurgical tasks

- Drilling: Using a 4 mm diamond burr, participants removed a 1 cm–diameter circular area of the calcium layer from a raw hen’s egg fixed in an egg cup, avoiding perforation of the underlying membrane. Time and procedural accuracy (membrane integrity, complete removal of calcium) were evaluated.
- Neuronavigation: A cashew nut embedded in agar within a plastic container was placed inside a 3D-printed skull model. A cone-beam CT scan was performed, and neuronavigation accuracy was verified by matching the navigation software output with anatomical landmarks (the Brainlab® software packages used included Cranial 3.1, with the following modules: Automatic Registration 2.5, Cranial Navigation 4.1, and Cranial Registration 3.6). Using a standard neurosurgical setup, participants were instructed to extract the nut en bloc with forceps along the shortest navigated trajectory by providing a triplanare reconstruction of a CT-scan displayed on a monitor, visualizing the exact position of the forceps and the target-lesion within the skull model. Time, completeness of retrieval, and number of adjustments were documented.

#### Other complex motor skill tasks

- Curtain Rod: Based on the experimental design described by Peter^13^ to assess alcohol-related impairment of motor coordination, participants were instructed to sequentially thread ten curtain rings onto a horizontally mounted rod. Performance was measured in terms of time and execution quality. Quality was assessed by recording the number of rings dropped and the number of failed attempts to hit the rod.
- Purdue Pegboard (model 32020 A, Lafayette instrument, US)^14^: Pins were inserted into designated holes on a pegboard under various conditions: unimanual (dominant and non-dominant hand), bimanual, and sequential assembly of a pin, washer, collar, and another washer. The number of successful placements in the respective time intervals (30 and 60 s) was evaluated.

All stations were supervised by the same specialist, experienced in the respective field (neurosurgery, forensic medicine and neuropsychology), to minimize observer bias. With the exception of the assessment of macroscopic and microscopic suture quality, which was rated using a 1–5 scale, no subjective performance evaluation was involved. Performance assessment was primarily based on objective outcome measures, including completion time, binary outcomes (e.g., whether the egg membrane was perforated: yes/no), and numerical measures (e.g., number of egg membrane defects). The task supervisor was responsible for explaining the tasks, monitoring task execution, and documenting performance outcomes. Participants were instructed to perform each task as quickly as possible, with an emphasis on error-free execution. The supervisor was not blinded to participant condition, estimated BAC, or the study hypothesis.

### Determination of BAC

Blood samples were analyzed for ethanol concentration at the Institute of Legal Medicine in Essen in accordance with the German forensic guidelines for alcohol analysis. Four independent determinations were performed using both gas chromatography with a flame ionization detector and enzymatic (alcohol dehydrogenase) method. The BAC was calculated as the arithmetic mean of these four concentrations^15^.

### Ethics statement

Ethical approval for this study was granted by the ethics committee of the University of Duisburg-Essen, Germany (approval number 23-11469-BO). The study was performed in accordance with the Declaration of Helsinki and all relevant guidelines and regulations. Participation was voluntary, and all participants provided written informed consent before participating in the study. All data were anonymized before analysis.

### Statistical analysis

Given the small sample size, normality was not assumed and nonparametric tests were used. Changes in binary outcomes across three time points were assessed with the exact two-sided Cochran’s Q test, with post hoc Bonferroni adjustment. Odds ratios (ORs) and 95% confidence intervals (CIs) were calculated using the Haldane–Anscombe correction (adding 0.5 to all cells) to address zero counts. Changes in continuous outcomes across three time points were analyzed with the two-sided Friedman test, with post hoc Bonferroni adjustment. Based on calculated values, pairwise comparisons were included.

To assess differences between the measurement time points T0, T1, and T2, median values across all participants were calculated and subjected to either a two-sided Friedman test or Cochran’s Q test, each with Bonferroni correction applied. Significant differences between test results were identified in a substantial number of tasks across all three test categories. For each individual task, we subsequently determined whether participants performed better, worse, or remained unchanged at T1 and/or T2 compared with T0, analyzed separately for the three test categories.

Analyses were conducted in IBM SPSS Statistics version 30.0.0.0 with α = 0.05 (two-tailed). Figures were created in GraphPad Prism version 10.4.1.

## Results

### Study cohort

Nine neurosurgeons (7 male, 2 female) participated in the study, and performed a total of 27 trials, resulting in 189 evaluable tests. The mean age was 32 years (range: 26–37), with professional experience in a surgical specialty ranging from 0.6 to 6 years (median 3.6 years). According to the AUDIT results, seven participants reported low-risk alcohol consumption, whereas two participants showed indications of at-risk drinking.

### BAC

The blood samples collected after the first run (baseline) showed a BAC of 0.0 g/kg for all participants. Following alcohol consumption, the median BAC was 0.96 g/kg, ranging from 0.73 g/kg to 1.12 g/kg. The minimum BAC resulted from one participant not completing the entire calculated amount of alcohol. During the elimination phase, the median BAC was 0.64 g/kg, ranging from 0.47 to 0.74 g/kg.

### Task performance

**Fig. 2:**
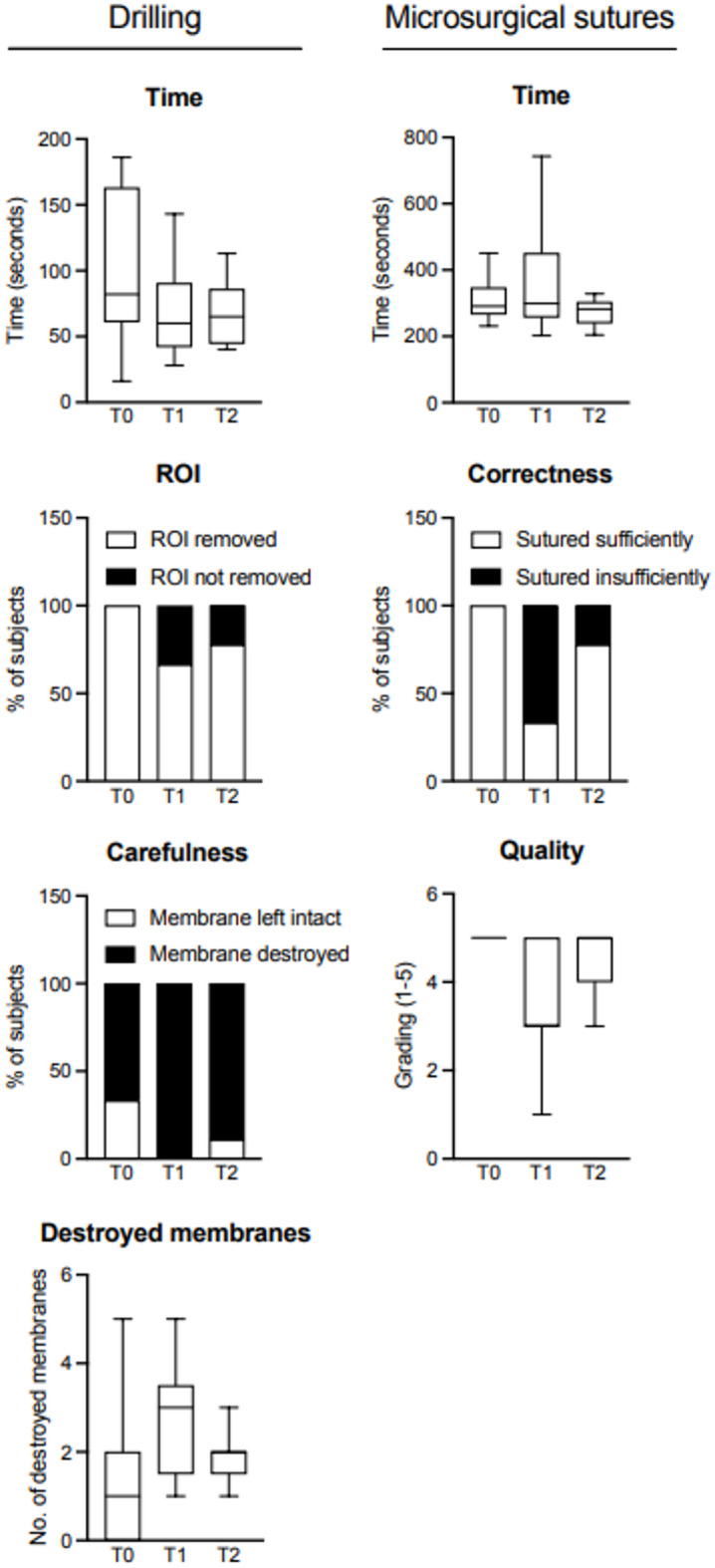
Graphical representation of test results in the category “Precise motor skills”. Results are shown for the test series “Drilling” and “Microsurgical sutures”. Metric data are presented as box-and-whisker plots, while categorical data are displayed as bar charts (%). Performances at all three time points (T0, T1, and T2) are depicted. Abbreviations: ROI = Region of interest.

**Fig. 3:**
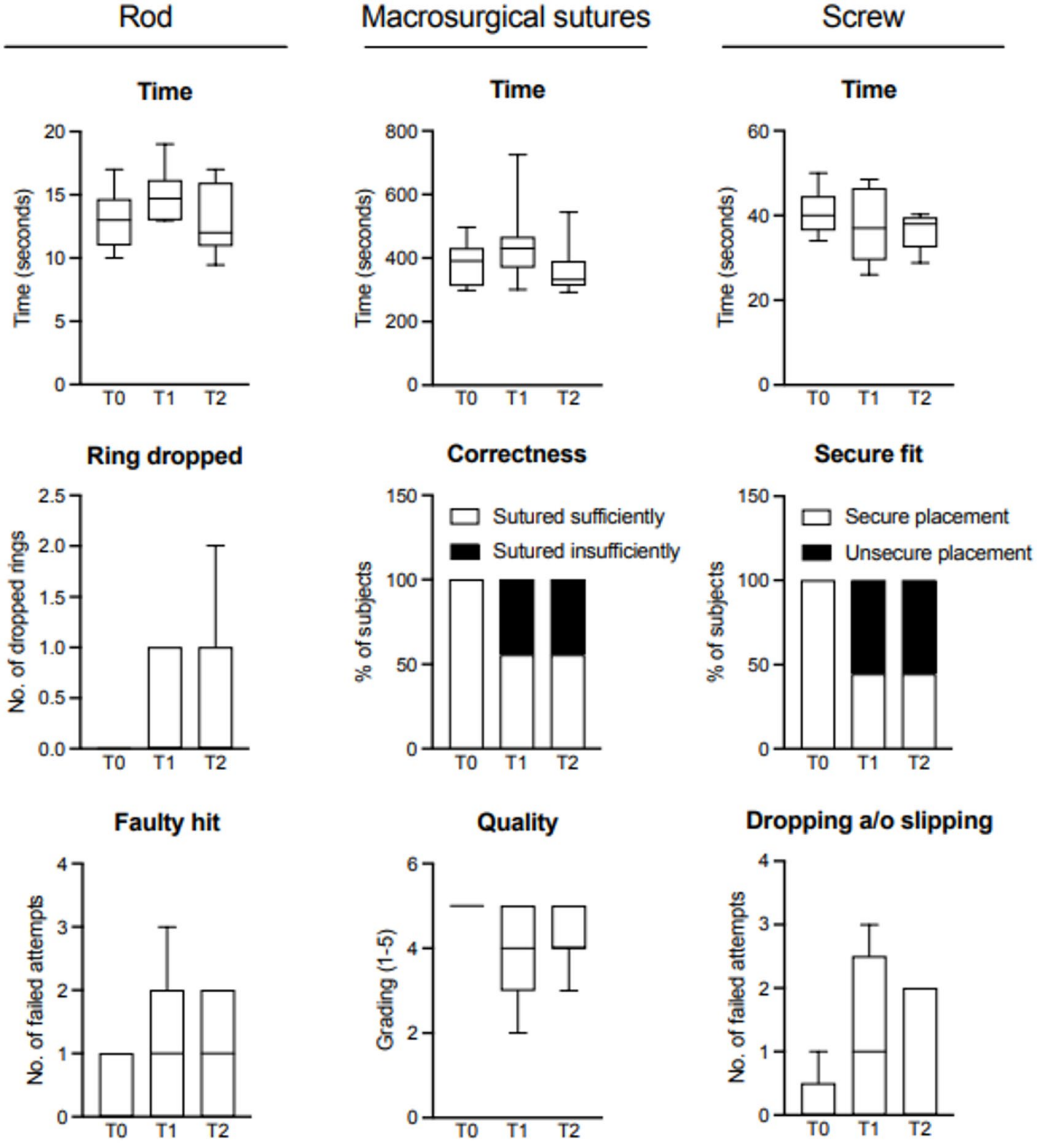
Graphical representation of test results in the category “Major motor skills”. Results are shown for the test series “Rod”, “Macro-surgical sutures”, and “Screw”. Metric data are presented as box-and-whisker plots, while categorical data are displayed as bar charts (%). Performances at all three time points (T0, T1, and T2) are depicted. Abbreviations: a/o = and/or.

**Fig. 4:**
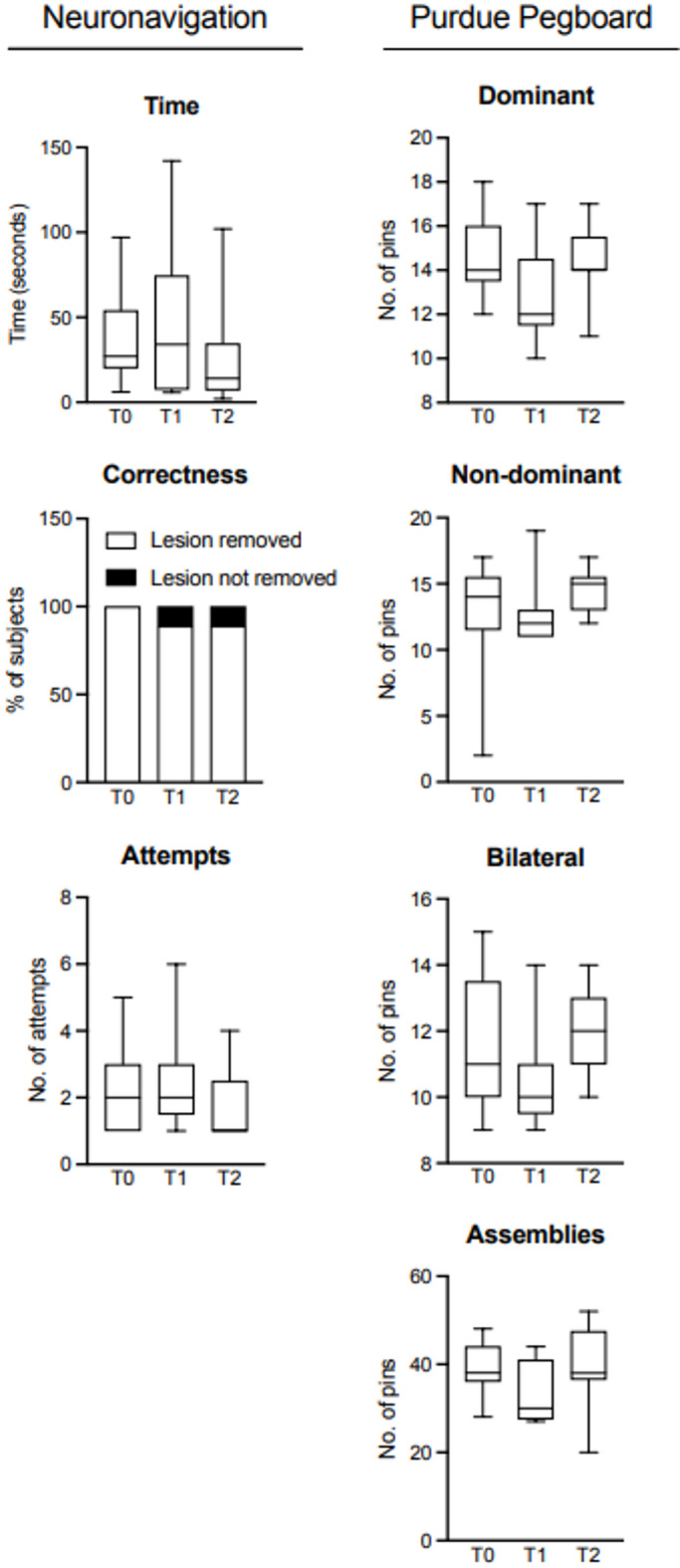
Graphical representation of test results in the category “Coordination”. Results are shown for the test series “Neuronavigation” and “Purdue Pegboard”. Metric data are presented as box-and-whisker plots, while categorical data are displayed as bar charts (%). Performances at all three time points (T0, T1, and T2) are depicted.

The results for the entire cohort are presented in Table 1, separated into precise motor skills (including the drilling and microsurgical suture tasks; Fig. 2), major motor skills (including the curtain rod, macrosurgical suture, and screwing tasks; Fig. 3), and coordination (including the neuronavigation and Purdue Pegboard tasks; Fig. 4).

**Table 1:** Differences in performance between baseline (T0) and assessments under alcohol consumption (T1, T2). Statistically significant p values are shown in bold. Abbreviations: A = two-sided Friedman test with Bonferroni correction across three time points; B = two-sided Cochran’s Q test with Bonferroni correction across three time points; IQR = interquartile range; M = median; N = number; ROI = region of interest.

	T0	T1	T2	p
*Precise motor skills*				
Drilling				
Time [M (IQR)]	106 (71–163)	60 (42–90.5)	65 (44.5–86)	**.013**^**A**^
ROI removed [N (%)]	9 (100)	6 (66.7)	7 (77.8)	.174^B^
Membrane intact [N (%)]	3 (33.3)	0 (0)	1 (11.1)	.097^B^
Perforations [M (IQR)]	1 (0–2)	3 (1.5–3.5)	2 (1.5–2)	.092^A^
Microsurgical sutures				
Time [M (IQR)]	291 (266–347)	299 (257–451)	282 (239.5–303.5)	.459^A^
Sufficiently sutured [N (%)]	9 (100)	3 (33.3)	7 (77.8)	**.009**^**B**^
Rating [M (IRQ)]	5 (5–5)	3 (3–5)	5 (4–5)	**.008**^**A**^
*Major motor skills*				
Rod				
Time [M (IQR)]	13 (11–14.7)	14.7 (13–16.2)	12 (11–15.9)	.247^A^
Ring dropped [M (IQR)]	0 (0–0)	0 (0–1)	0 (0–1)	.074^A^
Faulty hit [M (IQR)]	0 (0–1)	1 (0–2)	1 (0–2)	**.047**^**A**^
Macroscopic sutures				
Time [M (IQR)]	391 (313–431)	430 (370–466)	332 (313.5–390)	**.045**^**A**^
Sufficiently sutured [N (%)]	9 (100)	5 (55.6)	5 (55.6)	.069^B^
Rating [M (IQR)]	5 (5–5)	4 (3–5)	4 (4–5)	**.032**^**A**^
Screw				
Time [M (IQR)]	40 (36.5–44.5)	37 (29.5–46.6)	38 (32.5–39.5)	.107^A^
Secure fit [N (%)]	9 (100)	4 (44.4)	4 (44.4)	**.028**^**B**^
Dropping a/o slipping [M (IQR)]	0 (0–.5)	1 (0–2.5)	0 (0–2)	.086^A^
Coordination				
Neuronavigation				
Time [M (IQR)]	27 (20–54)	34 (7.5–74.5)	14 (7–34.5)	.459^A^
Lesion removed [N (%)]	9 (100)	8 (88.9)	8 (88.9)	.607^B^
Attempts [M (IQR)]	2 (1–3)	2 (1.5–3)	1 (1–2.5)	.607^A^
Purdue Pegboard				
Dominant [M (IQR)]	14 (13.5–16)	12 (11.5–14.5)	14 (14–15.5)	.140^A^
Non-dominant [M (IQR)]	14 (12–15.5)	12 (11–13)	15 (13–15.5)	**.029**^**A**^
Bilateral [M (IQR)]	11 (10–13.5)	10 (9.5–11)	12 (11–13)	**.018**^**A**^
Assemblies [M (IQR)]	38 (36–44)	30 (27.5–41)	38 (36.5–47.5)	.065^A^

Following alcohol consumption (phase of acute alcohol influence with an approximate BAC of 1.0 g/kg), the median duration for most tasks was slightly shorter than in the baseline (sober) condition, as illustrated in Fig. 2. However, tasks with higher demands on coordination and fine motor skills—such as suturing, neuronavigation, and the curtain rod task—required more time to complete (Figs. 3, 4). Despite occasional improvements in execution speed, overall performance quality declined across all tasks under acute alcohol influence (Figs. 2, 3, 4).

In the elimination phase (4–5 h after onset of alcohol consumption, with an approximate BAC of 0.6 g/kg), all tasks showed a shorter median duration compared to the baseline (sober) condition. With the exception of drilling and screw removal, median durations were also shorter than during the acutely influenced phase. Although performance quality showed partial improvements compared to the acute phase, it remained below the level observed in the sober condition in all tasks (Figs. 2, 3, 4).

Baseline performance (T0) showed only minor inter-individual differences, mainly in task completion time. To account for potential effects of baseline skill differences on performance outcomes, both absolute performance and intra-individual performance changes across conditions were evaluated.

**Fig. 5:**
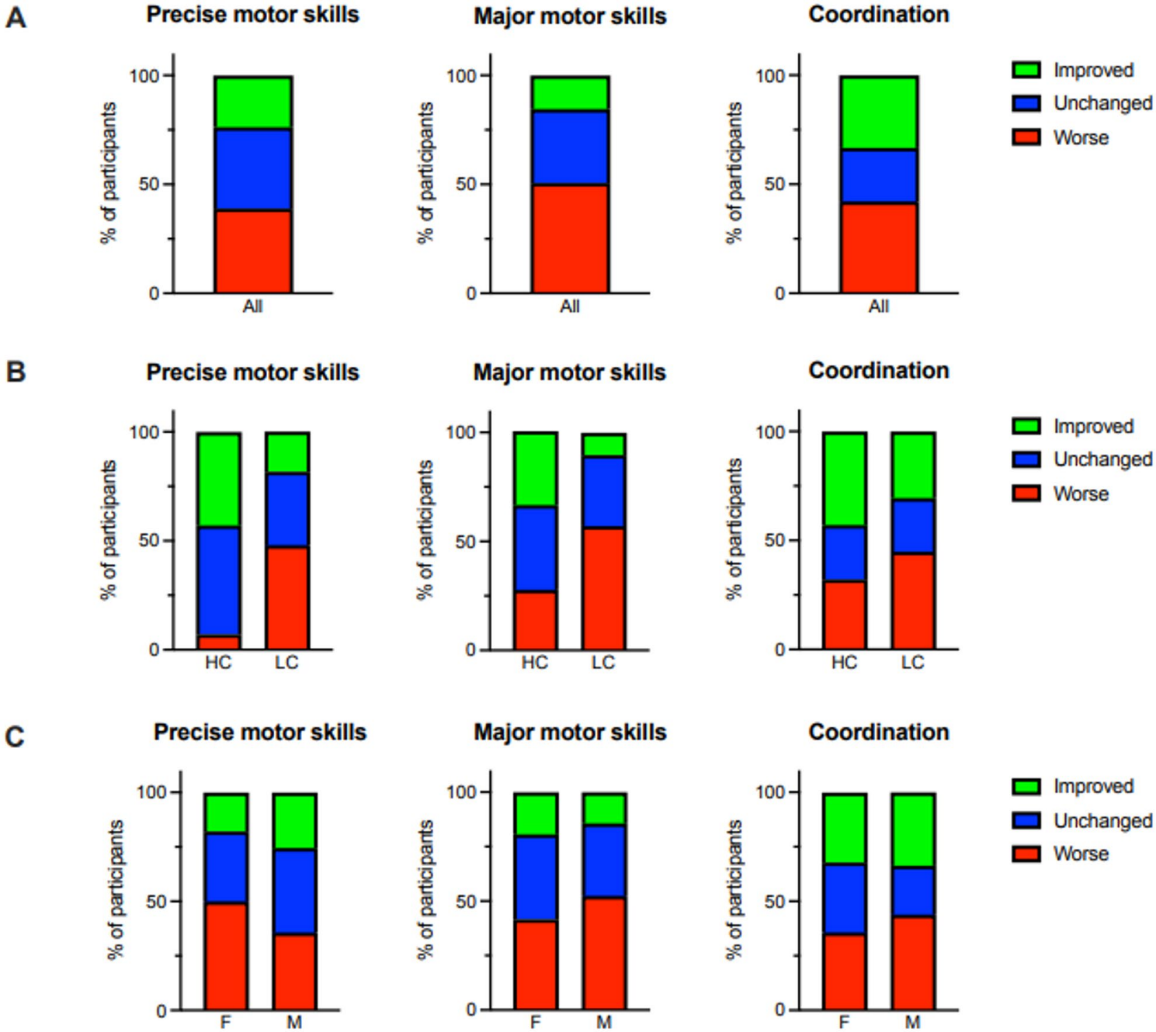
Graphical summary of test results in distinct subgroups. (**A**) All participants were examined across three domains: precise motor skills, major motor skills, and coordination. For each domain, performance at T1 and T2 was classified relative to baseline T0 as unchanged, improved, or worse. (**B**) Cohorts with low (LC) and high (HC) habitual alcohol consumption were compared across three domains: precise motor skills, major motor skills, and coordination. For each domain, performance at T1 and T2 was classified relative to baseline T0 as un-changed, improved, or worse. (**C**) Female (F) and male (M) participants were compared across three domains: precise motor skills, major motor skills, and coordination. For each domain, performance at T1 and T2 was classified relative to baseline T0 as unchanged, improved, or worse. Note: event counts are shown as the percentage of all eligible observations (i.e., % of possible events). Abbreviations: F = female; HC = high consumption; LC = low consumption; M = male.

Intra-individually, in the category of precise motor skills, 37.3% of results remained unchanged, 38.9% worsened, and 23.8% improved; in major motor skills, 34.0% remained unchanged, 50.6% worsened, and 15.4% improved; and in coordination tasks, 24.6% remained unchanged, 42.1% worsened, and 33.3% improved (Fig. 5A; Supplementary Fig. 1). Improvements were observed primarily in time-related parameters, whereas enhancements in task quality were rare.

Given the evident heterogeneity in individual performance changes, the cohort was stratified into subgroups based non-risk versus at-risk alcohol consumption (Fig. 5B; Supplementary Fig. 1) and sex (Fig. 5C; Supplementary Fig. 1). Participants with higher alcohol consumption demonstrated a greater proportion of improved or unchanged results, whereas these differences did not appear to be attributable to sex or to other potentially relevant distinctions between the high- and low-consumption groups (Supplementary Fig. 1). Owing to the small sample size, the subgroup analysis was restricted to descriptive reporting, and no statistical subgroup testing was performed.

## Discussion

In a controlled drinking trial, nine neurosurgical residents performed visuo-manual surgical and neurosurgical tasks in three conditions: sober, in the phase of acute alcohol influence (~ 1.0 g/kg) and in the alcohol elimination phase (~ 0.6 g/kg). It was hypothesized that performance would be impaired under the influence of alcohol.

In line with our hypothesis, a deterioration in surgical skill quality was observed across all 126 task performances under the influence of alcohol, compared to the sober baseline. During acute influence, only one third of all microsurgical sutures were rated as sufficient. Even several hours after alcohol consumption, only 55% of macroscopic sutures met predefined quality standards. In neurosurgery-specific tasks, no egg membrane remained intact, and fewer nuts were retrieved under intoxicated conditions. These findings support the recommendation that no surgical procedures should be performed under the influence of alcohol, not even several hours after moderate consumption.

While performance quality declined, task completion time partially improved, particularly in less complex procedures. This paradoxical effect was found in many previous studies^16–20^ and may reflect alcohol-related disinhibition, where cognitive impairment leads to increased self-confidence and risk-taking behavior, resulting in faster but often less accurate task execution^21,22^.

In previous research, similar alcohol-related impairments on surgical performance have been reported. Dorafshar et al.^23^ assessed 28 medical students in a single-blinded, prospective, controlled trial investigating simulated laparoscopic performance. Fourteen students were alcohol-impaired, and 14 were assigned to a placebo condition. The performance of the alcohol-impaired group (mean BAC 0.85 g/kg) significantly deteriorated in both time and quality measures 60–90 min after alcohol consumption, while no hangover effect was observed 10 h later.

In contrast, Gallagher^24^ and Van Dyken^8^ showed that alcohol intake on the evening before assessment led to impaired laparoscopic performance the next day, implying residual cognitive–motor impairment. Consistent with this, Hartung et al.^25^ reported that hangover effects during routine motor tasks like riding a bike may lead to performance decrements comparable to those observed at a BAC of approximately 0.3 g/kg—even in the absence of measurable alcohol levels.

A further study by Vasankari et al.^10^ did not assess (simulated) laparoscopic skills but instead evaluated fine-motor performance in six neurosurgeons. Aside from the present study, this is the only investigation that focuses exclusively on neurosurgeons rather than on surgeons from other specialties. Participants were required to suture a latex glove, as well as a latex tube under the operating microscope. Suturing was repeated after each of four doses of alcohol, resulting in a median breath alcohol concentration of 0.44 g/kg after the final dose. While small amounts of alcohol did not impair microsurgical suturing performance, the quality of macroscopic sutures declined with increasing alcohol concentration, and all suturing tasks were performed more quickly as BAC levels rose. Zyluk et al.^26^, however, could not find any significant differences in the duration of suturing a latex glove after 0.5 L of beer in medical students.

On an intraindividual level, participants with alcohol intake several times per week appeared to show fewer alcohol-related performance impairments. This could potentially reflect habituation effects as shown in previous studies^27,28^; however, this interpretation must be treated with caution, as the observation is based on only two individuals which is far too small to draw any reliable conclusions.

Notably, a subset of participants showed improved performance compared with baseline, including 23.8% in fine motor tasks, 15.4% in gross motor tasks, and 33.3% in coordinative tasks. Although all participants completed a non-scored familiarization run prior to baseline assessment to reduce learning effects, repeated task execution may still have contributed to performance improvements. Furthermore, classification as improved performance was based not only on quality-related measures but also on task completion time. Therefore, apparent improvements may partly reflect alcohol-related disinhibition resulting in faster task execution rather than a true improvement in performance quality.

## Limitations and strengths

Several limitations apply to this study. First, the small sample size limits both the statistical power and the generalizability of the findings. Moreover, testing under the influence of substances is often limited by the artificial nature of laboratory settings, in which participants may be especially motivated to perform at their best. This can raise questions regarding the real-life applicability of such findings. In the present study, however, the scenario closely reflects the likely behavior of a surgeon under the influence of alcohol, who would still aim to achieve the best possible surgical outcome despite the impaired state. Therefore, the transferability of the observed performance to clinical reality appears to be generally justified. However, the absence of a repeatedly assessed sober control group and the repeated execution of tasks within the study period may have introduced learning or adaptation effects, even though most of the procedures performed can be considered routine. A sober control group assessed at the same time points would have allowed better differentiation between alcohol-related effects and practice-related performance improvements. Future studies should incorporate repeated sober assessments to address this limitation. As food intake was not standardized or monitored it may have contributed to inter-individual variability in alcohol absorption and performance. Food intake influences gastric emptying, a delayed gastric emptying may reduce peak BAC and attenuate absorption-phase effects. However, this effect is mainly expected after heavy, high-fat meals rather than the light food permitted in this study. The achieved median BAC (0.96 g/kg) being close to the calculated target BAC (1.0 g/kg) suggests a very limited impact at the group level. Moreover, performance at the lower BAC level was assessed only during the elimination phase where less alcohol-related effects are expected (Mellanby effect^29^). Therefore, it seems appropriate to assume that surgical skill quality would have been worse at comparable BACs during the phase of alcohol absorption. Finally, the study focused on manual surgical skills and did not assess other critical competencies such as surgical planning, intraoperative decision-making, or team-based communication.

Nevertheless, the participants underwent thorough testing and were required to complete several distinct tasks that demanded a broad range of surgical skills. Each test series consisted of 7 tests, leading to a complex profile. In contrast to many previous studies, the experimental setup was highly realistic, employing pig skin for suturing and a three-dimensional skull model rather than computer simulations. Another strength of the study is that the evaluation focused not only on task completion time and performance quality, but also on intraindividual effects, thereby allowing a more nuanced and comprehensive assessment of surgical proficiency.

## Conclusion

The findings of this study highlight the potential risks associated with alcohol-impaired surgical performance. Further research is warranted to investigate the interaction between alcohol consumption and surgical proficiency, particularly regarding hangover effects and the residual impact of substances such as alcohol, cannabis, and other drugs. Studies involving larger and more diverse cohorts—including highly experienced and alcohol-habituated surgeons—are required to clarify which variables may attenuate substance-related performance deficits. Future studies should also incorporate repeatedly assessed sober control groups to better distinguish substance-related effects from practice-related improvements. Overall, the findings of this study suggest that substance-related impairment may affect surgical performance even in trained professionals, with potential implications for patient safety.

## Supplementary Information

Supplementary Information.

## Data Availability

The datasets generated during the current study are available from the corresponding author on reasonable request.
