# Supplementary material for: Impact of moderate alcohol consumption on visuo-motor skills in neurosurgical residents
Source: Sci Rep. 2026 Jun 16;16:18691. doi: 10.1038/s41598-026-58320-5 (PMC13272685; doi:10.1038/s41598-026-58320-5)
Supplement: Supplementary file 1 — Supplementary Information. [file 41598_2026_58320_MOESM1_ESM.docx]

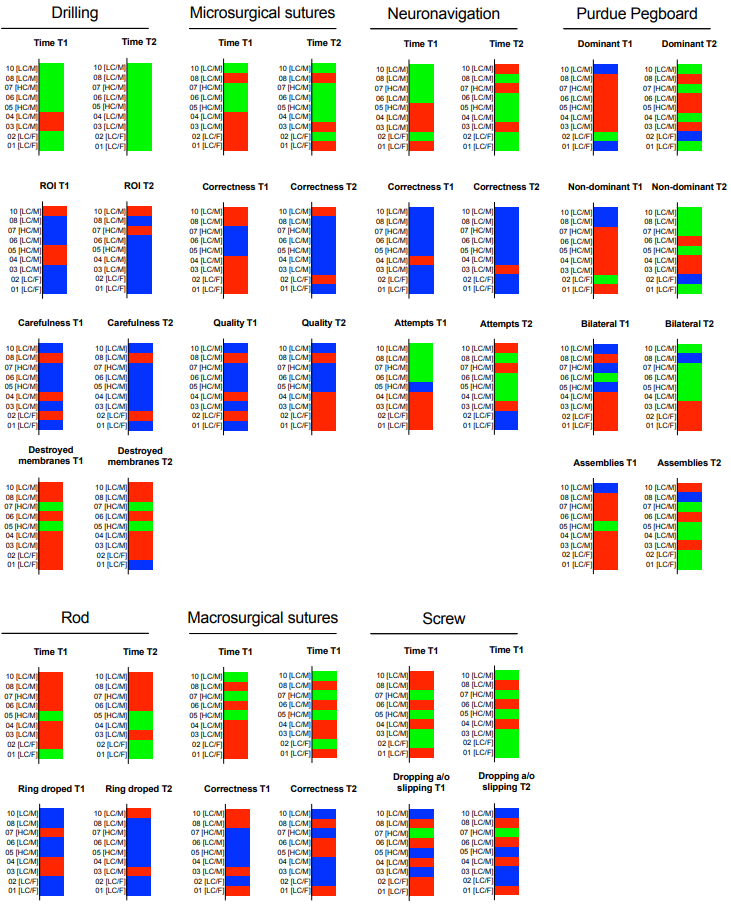


**Supplementary Figure 1.** Test results of all participants are displayed. The figure illustrates whether participants achieved outcomes at T1 and T2 that were comparable to baseline T0 (indicated in blue), improved (indicated in green), or worsened (indicated in red). Abbreviations: F = female; HC = high consumption; LC = low consumption; M = male.
